# Objective evaluation of intracochlear electrocochleography: repeatability, thresholds, and tonotopic patterns

**DOI:** 10.3389/fneur.2023.1181539

**Published:** 2023-08-08

**Authors:** Klaus Schuerch, Wilhelm Wimmer, Christian Rummel, Marco Domenico Caversaccio, Stefan Weder

**Affiliations:** ^1^Department of ENT, Head and Neck Surgery, Inselspital, Bern University Hospital, University of Bern, Bern, Switzerland; ^2^Hearing Research Laboratory, ARTORG Center for Biomedical Engineering Research, University of Bern, Bern, Switzerland; ^3^Department of Otorhinolaryngology, TUM School of Medicine, Klinikum Rechts der Isar, Technical University of Munich, Munich, Germany; ^4^Support Center for Advanced Neuroimaging (SCAN), University Institute for Diagnostic and Interventional Neuroradiology, Inselspital, Bern University Hospital, University of Bern, Bern, Switzerland

**Keywords:** ECochG, deep learning, electrophysiology, cochlear implants, hearing loss, signal processing, electric acoustic stimulation, hearing preservation

## Abstract

**Introduction:**

Intracochlear electrocochleography (ECochG) is increasingly being used to measure residual inner ear function in cochlear implant (CI) recipients. ECochG signals reflect the state of the inner ear and can be measured during implantation and post-operatively. The aim of our study was to apply an objective deep learning (DL)-based algorithm to assess the reproducibility of longitudinally recorded ECochG signals, compare them with audiometric hearing thresholds, and identify signal patterns and tonotopic behavior.

**Methods:**

We used a previously published objective DL-based algorithm to evaluate post-operative intracochlear ECochG signals collected from 21 ears. The same measurement protocol was repeated three times over 3 months. Additionally, we measured the pure-tone thresholds and subjective loudness estimates for correlation with the objectively detected ECochG signals. Recordings were made on at least four electrodes at three intensity levels. We extracted the electrode positions from computed tomography (CT) scans and used this information to evaluate the tonotopic characteristics of the ECochG responses.

**Results:**

The objectively detected ECochG signals exhibited substantial repeatability over a 3-month period (bias-adjusted kappa, 0.68; accuracy 83.8%). Additionally, we observed a moderate-to-strong dependence of the ECochG thresholds on audiometric and subjective hearing levels. Using radiographically determined tonotopic measurement positions, we observed a tendency for tonotopic allocation with a large variance. Furthermore, maximum ECochG amplitudes exhibited a substantial basal shift. Regarding maximal amplitude patterns, most subjects exhibited a flat pattern with amplitudes evenly distributed over the electrode carrier. At higher stimulation frequencies, we observed a shift in the maximum amplitudes toward the basal turn of the cochlea.

**Conclusions:**

We successfully implemented an objective DL-based algorithm for evaluating post-operative intracochlear ECochG recordings. We can only evaluate and compare ECochG recordings systematically and independently from experts with an objective analysis. Our results help to identify signal patterns and create a better understanding of the inner ear function with the electrode in place. In the next step, the algorithm can be applied to intra-operative measurements.

## 1. Introduction

Electrocochleography (ECochG) is increasingly being used to measure residual inner ear function in cochlear implant (CI) recipients. This facilitates the direct recording of signals from the implant electrode array either during or at any time after implant surgery. Using the ECochG signals, we can map and monitor the inner ear tonotopic and temporary function. ECochG is an umbrella term for four different inner ear potentials (i.e., cochlear microphonic CM, auditory neurophonic ANN, compound action potential CAP, and summating potential SP) (1–3). The CM/DIF response is the most common in the analysis of inner ear function because it is the most reliable and robust cochlear signal (4). It was calculated by subtracting the responses to the condensation (CON) and rarefaction (RAR) polarity stimuli.

The signal characteristics of the ECochG measurements provide different insights into the cochlear function. Several publications have reported that abrupt drops in the CM/DIF amplitudes during cochlear implantation may be associated with traumatic inner ear events (5–8). In a post-operative setting, CM/DIF signals exhibited a strong correlation with audiometric hearing thresholds (9, 10). Furthermore, the CM/DIF amplitudes varied across the electrode carrier, depending on the recording site. In contrast to the assumption that the amplitude peaks at the tonotopic position for a given acoustic stimulus (11–17), different amplitude patterns along the cochlear duct have been described (i.e., basal and flat amplitude patterns) (11, 12, 14).

Until now, the diagnostic gold standard for analyzing ECochG signals has been expert visual inspection. However, this approach entails several limitations. Visual interpretation depends on experts and requires experience in the field. Hence, reproducibility is limited, longitudinal data can only be assessed to a limited extent, and different studies may reach different results, hampering direct comparisons. Moreover, if the signal-to-noise ratio (SNR) is poor, recordings are often not included in the analysis, leading to selection and reporting biases. In conclusion, a deeper and more systematic understanding of the signal behavior is needed before ECochG recordings can be used and interpreted in clinical routine. Therefore, new analytical approaches are required for this purpose.

In our previous study, we proposed machine-learning models that objectively identified CM/DIF signals (18). The Hotelling's *T*^2^ Test and Deep Learning (DL) approach yielded high accuracies. The proposed objectification methods make observations transparent because the analysis is always performed in the same manner. Furthermore, no data were neglected owing to a poor SNR avoiding a selection bias. Therefore, the objective analysis method facilitates the study and comparison of the longitudinal data and ECochG patterns.

The aim of the current study was to apply our objective algorithm and evaluate the repeatability and patterns of the intracochlear ECochG measurements. We tested three hypotheses: (i) longitudinal ECochG measurements remain stable with unchanged residual inner ear function (repeatability); (ii) ECochG thresholds correlate with hearing thresholds; and (iii) for different frequencies, CM/DIF amplitudes show their maxima at different intracochlear locations according to their tonotopic organization.

## 2. Methods

This prospective cohort study was conducted in accordance with the Declaration of Helsinki and approved by the local institutional review board (KEK-BE 2019-01578). Written informed consent was obtained from the individuals for the publication of any potentially identifiable data included in this article.

### 2.1. Data acquisition

We included 20 adults in our study (*n* = 21 ears; 12 females, eight males; mean age, 60.0 years; SD = 16.5 years, range, 25–82 years). All subjects received an implant from the same manufacturer (MED-EL, Innsbruck, Austria) at least 6 months prior to the study participation. This period is important to avoid rapid post-operative changes in the inner ear function, which occur predominantly within the first months after implantation (19).

**Figure 1:**
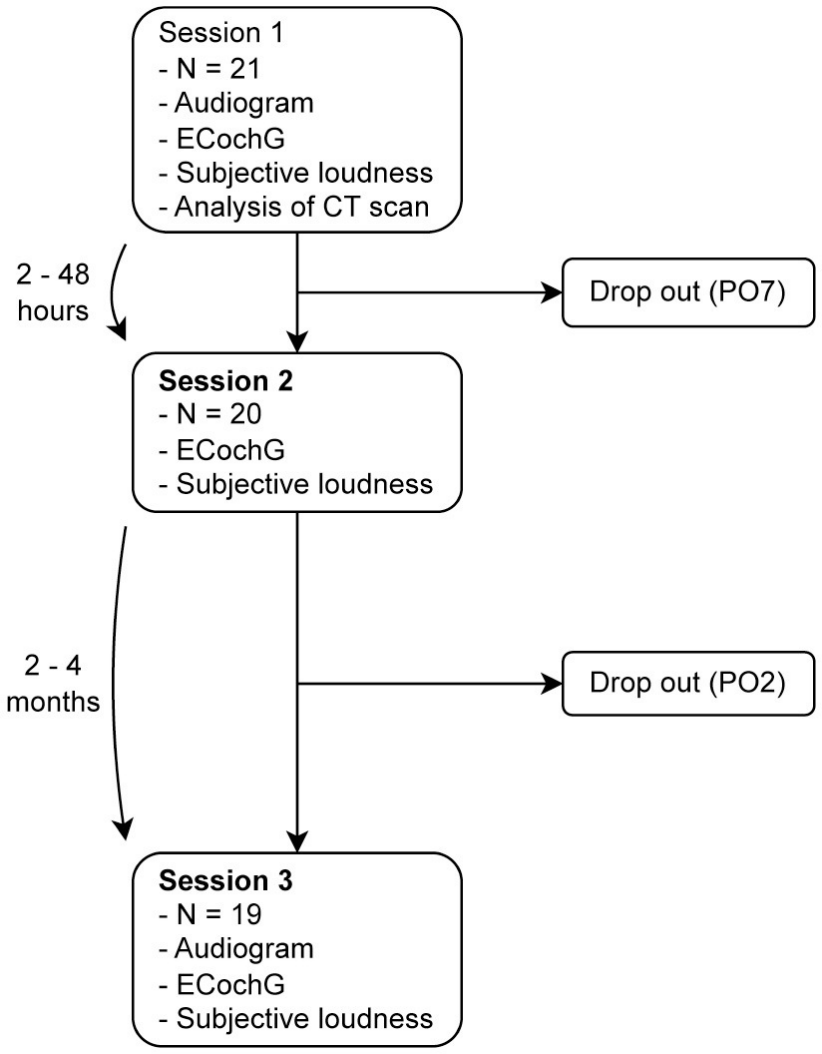
We performed three measurement sessions: (i) at least 6 months after implantation, (ii) within 2–48 h after the first measurement, and (iii) 2–4 months after the second measurement. Two subjects did not complete all the three sessions; one of them suffered from a complete hearing loss, so that no intracochlear signals could be measured, and the other withdrew from the study for personal reasons. PO, subject ID.

In total, we conducted three measurement sessions, as shown in Figure 1: (i) the first session was at least 6 months after implantation, (ii) the second session was 2–48 h after the first measurement, and (iii) the third session was 2–4 months after the second measurement. To obtain residual hearing, a pure-tone audiogram was performed at the beginning of sessions (i) and (iii) using a calibrated setup in a certified acoustic chamber (Interacoustics, Middlefart, Denmark). The subjects' audiograms are shown in the Supplementary material. According to Rasetshwane et al. (20), the subjects categorized the loudness of the acoustic ECochG stimuli into seven categories (not audible, very soft, soft, medium, loud, very loud, and too loud). The evaluated dataset is available at Schuerch et al. (21).

During the ECochG recordings, we used pure-tone stimuli at 250 Hz, 500 Hz, 750 Hz, 1 kHz, 1.5 kHz, and 2 kHz using the research Maestro software (version 8.03 AS and 9.03 AS, MED-EL, Innsbruck, Austria) (22). As a minimum requirement, we recorded the ECochG potentials at four electrodes (1, 4, 7, and 10) and three intensity levels (supra-threshold: 5 dB below the discomfort level, near-threshold: 10 dB above the pure-tone hearing threshold of the measured frequency, and sub-threshold: 10 dB below the pure-tone hearing threshold of the measured frequency). Additional adjacent intensity levels and electrodes were measured when the time permitted and when the subject agreed. For each electrode, the intensity level, frequency, and 100 epochs of each polarity (CON/RAR) were measured, digitized at 120 kHz, and stored separately.

In all but one subject (ID PO8), a routinely performed post-operative computed tomography (CT) scan of the temporal bone was available, from which we calculated the intracochlear electrode positions and their corresponding theoretical tonotopic frequencies using Otoplan software (version 3, CASCINATION, Bern, Switzerland). For the subject ID PO8, we used the average insertion depths obtained from 57 subjects using Flex28 electrodes (23).

### 2.2. Data analysis

#### 2.2.1. Preprocessing

To test our three hypotheses, we focused on the CM/DIF signal. We preprocessed the ECochG data as described in Schuerch et al. (18). We used the following steps: (i) removal of stitching artifacts; (ii) application of a Gaussian-weighted averaging method to remove uncorrelated epochs (24, 25); (iii) calculation of the CM/DIF signal by subtracting the CON and RAR recordings (1, 3); and (iv) application of a bandpass filter (100 Hz/5 kHz) (4). The signal-to-noise ratio (SNR) was calculated using the plus-minus averaging method (26). We obtained the SNR for each polarity separately and averaged both values to obtain the final SNR.

#### 2.2.2. Objectification

We used our previously described DL algorithm to classify the CM/DIF signals into “response present” and “response absent” subgroups (18). Using these categories, we tested three hypotheses: (i) repeatability, (ii) correlation with hearing thresholds, and (iii) frequency-specific amplitude maxima with respect to the tonotopic position.

#### 2.2.3. Repeatability

We analyzed the repeatability of the CM/DIF signals using prevalence-adjusted and bias-adjusted kappa (PABAK), which considers the prevalence and chance of agreement (27–29). Because PABAK and Cohen kappa were designed to compare only two variables (in our data: sessions), we computed them for all possible combinations (sessions: 1 and 2, 1 and 3, and 2 and 3). Finally, the kappa values across all three sessions were averaged to obtain the overall kappa value as proposed in (30). We evaluated the kappa values for three different intensity categories: (i) sub-threshold (intensity <0 dB), (ii) near-threshold (25 dB > intensity ≥ 0 dB), and (iii) supra-threshold (intensity ≥ 25 dB). The interpretation of the kappa values was based on that of Landis and Koch et al. (31). The kappa values were calculated using epiR (v. 2.0.52), R (v. 4.1.2), rpy2 (v. 3.5.4), and Python (v. 3.9.7) (32, 33). We tested whether the sessions matched by using McNemar's test and the Python Statsmodels module (v. 0.13.2) (34).

#### 2.2.4. Threshold analysis

We compared the objectively detected CM/DIF thresholds with the objective audiometry and subjective thresholds. First, we identified the relative stimulus intensities (ECochG stimulus-audiometric threshold) that produced objectively detected responses and non-responses, respectively. Second, for each stimulus type and subject, we determined the lowest relative stimulus intensity that still elicited an objective response. These values were compared to the pure-tone threshold and individual loudness perception. The objective responses elicited by higher acoustic intensities were neglected in this part of the analysis.

#### 2.2.5. Tonotopy and pattern analysis

For the tonotopic and pattern analysis, we used the measurement session with the most recordings for each subject. First, the CM/DIF amplitudes were normalized. A weighted mean was then calculated separately for all stimulation frequencies to assess the tonotopic distribution of the signal amplitudes. The weighted mean was calculated as follows:


$$\begin{array}{l} \bar{X}=\frac{\sum_{i=1}^{n}w_{i}x_{i}}{\sum_{i=1}^{n}w_{i}} \end{array}$$

where $\bar{X}$ is the weighted mean, *n* is the number of signals, *w* is the normalized amplitude, and *x* is the tonotopic position of the signal.

Finally, we checked for the presence of intracochlear CM/DIF patterns (that is, apical response, basal response, medial response, and flat response) similar to the previous findings by Bester et al. (11, 12). We integrated the frequency allocations from Li et al. (35) and divided them into the following tonotopic regions: apical (20–500 Hz), medial (500–4,000 Hz), and basal (4,000–20,677 Hz). We defined our patterns based on these frequency regions. We assigned a pattern of the maximum CM/DIF amplitude in one of the regions, which exceeded the median of all other recording locations by 30% or more for a given stimulus (11). We defined the “flat” pattern as in Bester et al. when multiple or no significant peaks from two or more tonotopic regions occurred (11).

## 3. Results

Table 1 lists the demographic characteristics of the subjects. The subjects most commonly had progressive hearing loss with a mean low frequency pure tone average (PTA at 125 Hz, 250 Hz, 500 Hz, and 1 kHz) of 88.7 dB HL (range: 39.0–113.8 dB HL). The subjects received either a Flex24 or Flex28 electrode with a mean insertion depth of 509° (range: 350° to 632°).

**Table 1:** Demographics of the 21 ears examined.

**Subj. ID**	**Gender**	**Age (years)**	**Side**	**Etiology**	**Electrode array**	**Insertion angle (°)**	**ToM (month)**	**PTA (dB HL)**
PO0	F	51–60	R	Progressive HL	Flex28	561	10	68.8
PO1	M	71–80	R	Progressive HL	Flex28	526	17	110.0
PO2	M	71–80	L	Progressive HL	Flex24	419	46	66.3
PO3	M	71–80	L	Congenital genetic	Flex28	524	9	85.0
PO4	F	21–30	R	Congenital genetic	Flex28	550	20	101.3
PO5	F	61–70	R	Progressive HL	Flex28	578	28	92.5
PO6	F	71–80	R	Meniere's disease	Flex28	536	78	90.0
PO7	M	81–90	L	Progressive HL	Flex28	547	75	113.8
PO8	F	21–30	R	Congenital genetic	Flex28	530	57	85.0
PO9	F	41–50	R	Progressive HL	Flex28	555	22	83.8
PO10	F	51–60	R	Progressive HL	Flex24	456	13	97.5
PO11	F	71–80	L	Progressive HL	Flex28	350	70	100.0
PO12	M	41–50	R	Meningitis	Flex28	564	11	81.3
PO13	F	61–70	L	Progressive HL	Flex28	526	22	93.8
PO14	F	51–60	R	Congenital genetic	Flex24	531	174	95.0
PO15	M	41–50	L	Meningitis	Flex28	538	6	75.0
PO16	M	61–70	R	Meniere's disease	Flex28	632	7	106.3
PO17	M	51–60	R	Sudden HL	Flex28	493	11	91.3
PO18	M	71–80	R	Progressive HL	Flex28	461	70	96.3
PO19	F	61–70	R	Progressive HL	Flex24	466	131	91.3
PO20	F	31–40	L	Progressive HL	Flex24	402	6	39.0
Mean		59.5				511.6	42.0	88.7

Pure-tone average (PTA) values in dB hearing level were calculated as the mean of the hearing thresholds at 125, 250, 500, and 1,000 Hz. Subj., subject; ToM, time of measurement in months after implantation; HL, hearing loss.

CM/DIF responses were detected in all subjects except for one who dropped out after the first session. Overall, the CM/DIF responses were detected in 27.5% of the signals (sub-threshold, near-threshold, and supra-threshold) and in 37.8% of the signals with acoustic stimulation above the hearing level (near-threshold and supra-threshold). The CM/DIF amplitudes ranged from 4.56 μV_pp_ to 74.46 μV_pp_. The sample recordings of CM/DIF, ANN/SUM, CON, RAR, and their individual Fast Fourier Transform (FFT) power spectra are shown in the Supplementary material (subject PO8).

### 3.1. Repeatability

**Figure 2:**
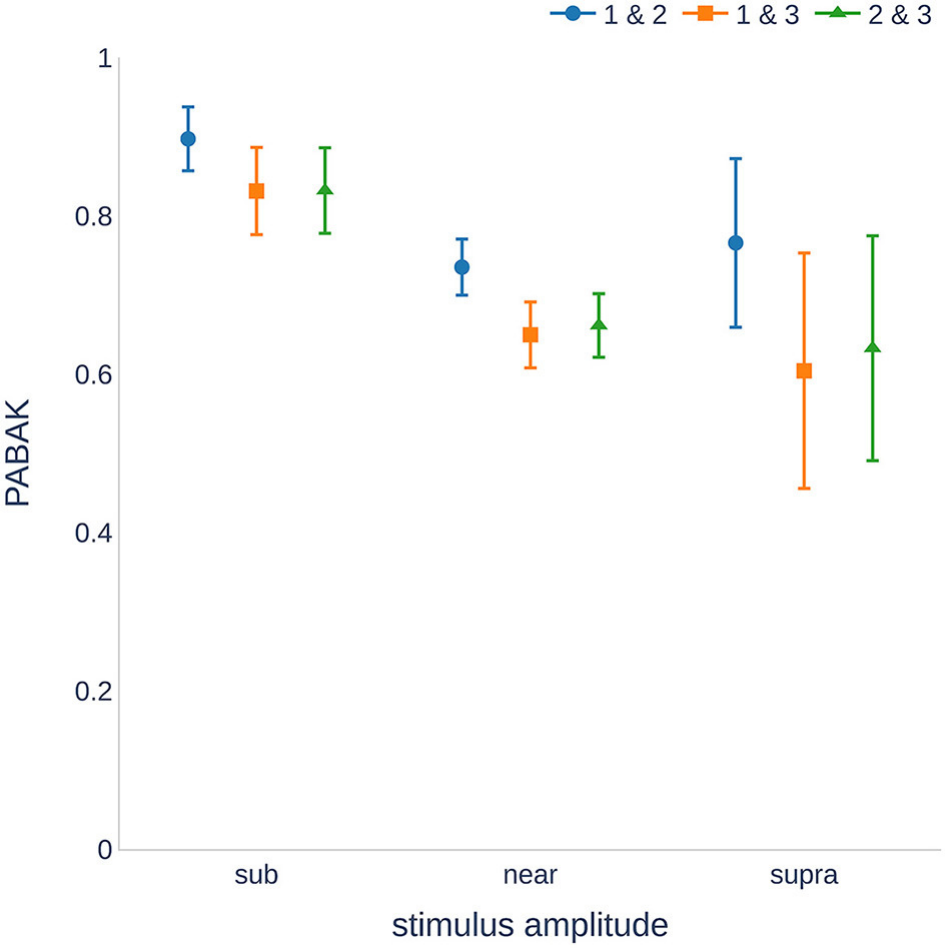
Reproducibility of the measurements ranged from substantial to almost perfect (29). The sub-threshold stimuli showed higher reproducibility than the near and supra-threshold stimulation. Higher variance was found for supra-threshold stimulation because fewer recordings were available. PABAK, prevalence-adjusted and bias-adjusted kappa; dB, decibel.

Our data showed substantial reproducibility across the three measurement sessions, as indicated by an average PABAK of 0.68 (Cohen's kappa coefficient of 0.61 and an accuracy of 84.1%). Figure 2 shows the PABAK values for combinations of the three recording sessions and three intensity levels (calculated as the difference between the stimulus level and individual hearing threshold).

The highest PABAK values were observed in the sub-threshold group. There was a wider confidence interval for the supra-threshold group because of the smaller number of recordings (stimulation of 25 dB above the hearing threshold was not possible for all individuals and frequencies). It should also be noted that the PABAK values for sessions 1 and 2 were consistently higher than those of the other session combinations. A further analysis of the sensitivity and specificity revealed that sessions 1 and 2 were not significantly different (*p* = 0.499, McNemar's test), whereas sessions 1 and 3, and 2 and 3 were significantly different (*p* = 0.009 and *p* < 0.001, respectively). Detailed information on the calculation of the kappa values can be found in the Supplementary material.

### 3.2. Thresholds

**Figure 3:**
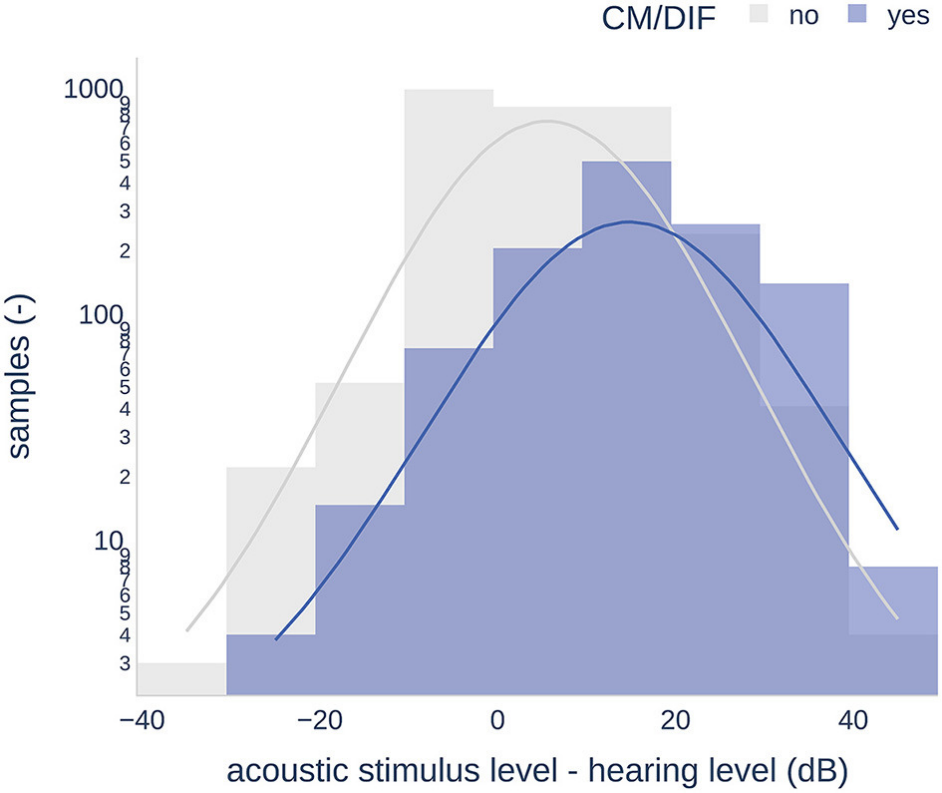
Histogram of objectively analyzed CM/DIF responses including all stimulation frequencies and intensity levels. Acoustic stimulus level-hearing level indicates the intensity of the stimulation relative to the individual's hearing threshold.

The mean PTAs were 88.7 dB HL (SD = 22.4) and 89.1 dB HL (SD = 20.9) for the first and third sessions, respectively. There were no significant differences between the two sessions (one-tailed paired-samples t-test, p = 0.096). Higher stimulation amplitudes generally resulted in a higher number of ECochG responses. Figure 3 shows a histogram of the CM/DIF recordings based on the relative stimulation level. It shows a considerable overlap between the distribution of the recordings with (blue shaded area) and without (gray shaded area) an objectively detected response. Both distributions showed a bell-shaped distribution, with large variances (range of ECochG response present: −30 to 48 dB, range of ECochG not present: −40 to 48 dB). The mean relative stimulation level for the recordings with a detected response was 14.1 dB (SD = 11.4), which was significantly higher than the mean relative stimulation level for the recordings without a detected response (3.5 dB, SD = 11.1, *p* < 0.001, one-tailed paired-samples *t*-test).

**Figure 4:**
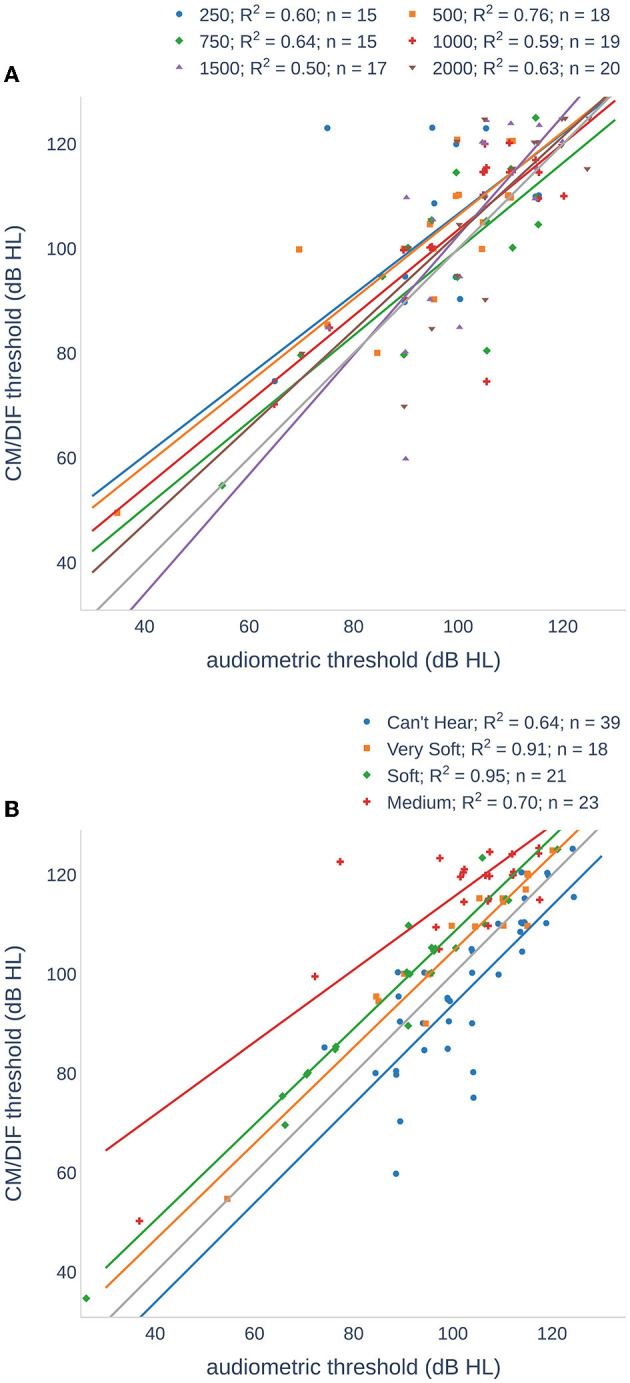
Scatter plots of CM/DIF thresholds as a function of audiometric thresholds for all subjects. **(A)** The CM/DIF thresholds were grouped according to their stimulus frequency. **(B)** The CM/DIF thresholds were grouped into four categories according to their subjective loudness: (i) can not hear (blue), (ii) very soft (orange), (iii) soft (green), and (iv) medium (red). The solid lines show the linear regression in both plots.

Figure 4 and Table 2 compare the individual ECochG thresholds with audiometric thresholds. We examined the frequency dependence of the CM/DIF and audiometric thresholds (Figure 4A) using linear regression models, which yielded *r*^2^ values between 0.50 and 0.76 (*p* < 0.001), indicating a moderate dependence between the two. Analyzing the same data in terms of subjects' perceived loudness (Figure 4B), we found *r*^2^ values between 0.64 and 0.95 (*p* < 0.001). The linear model fits the data best for very soft and soft perceptions.

**Table 2:** Mean and SD of the difference between audiometric and CM/DIF threshold.

	**CM/DIF vs. audiometric threshold (dB HL), our data**		**CM/DIF vs. audiometric threshold (dB HL), Koka et al**. **(****10****)**		**CM/DIF vs. audiometric threshold (dB nHL), Haumann et al**. **(****9****)**	
**Group**	**Mean**	**SD**	**Mean**	**SD**	**Mean**	**SD**
250 Hz	−8.5	15	−6	8	−12.0	17.5
500 Hz	−7.6	9.0	−6	8	5.9	11.8
750 Hz	−0.3	10.7	−4	9	
1 kHz	−3.3	9.7	0.3	9	23.0	11.4
1.5 kHz	−2.8	13.0	3	8	
2 kHz	−2.5	10.0	2	9	
Can't hear	6.2	9.0				
Very soft	−4.6	4.6				
Soft	−8.7	4.4				
Medium	−15.7	9.6				

SD, standard deviation.

### 3.3. Tonotopy and patterns

**Figure 5:**
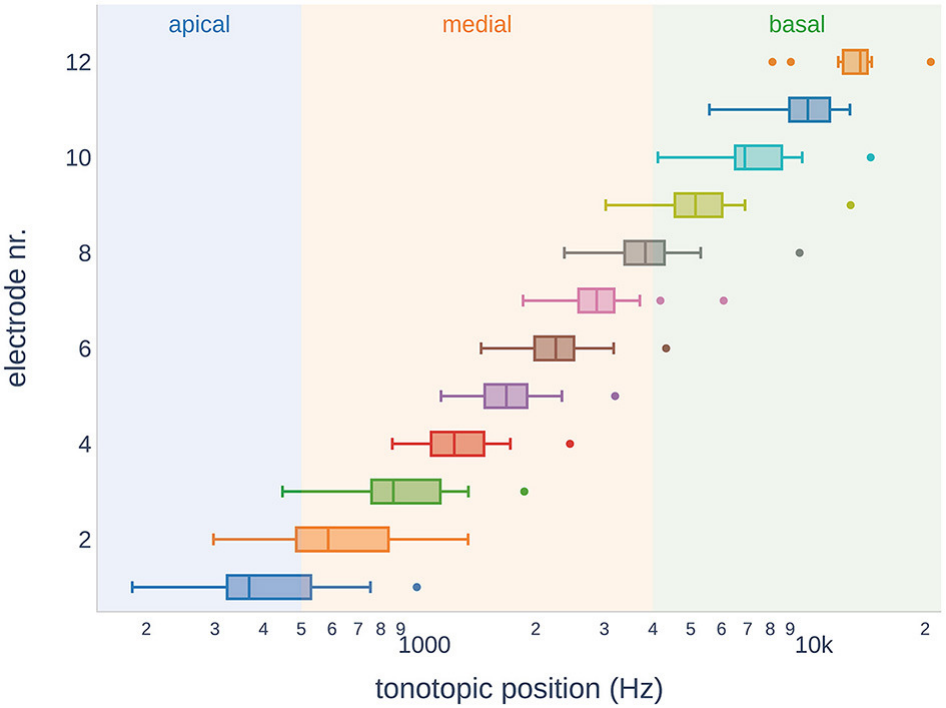
Tonotopic positions of the electrodes in the study subjects. Across the subjects, some electrodes showed overlap in the tonotopic region. It should be noted that not all electrode arrays reached the apical region of the cochlea.

Owing to the variable size of the cochlea in our study cohort, the insertion depth of the electrode varied considerably (ranging from 350° to 632°, see Table 1). Figure 5 shows the variance of the tonotopic positions of all the 12 electrodes. According to our cochlear frequency subdivision (i.e., apical, medial, and basal parts of the cochlea), not all electrodes reached the apical region (n = 6).

**Figure 6:**
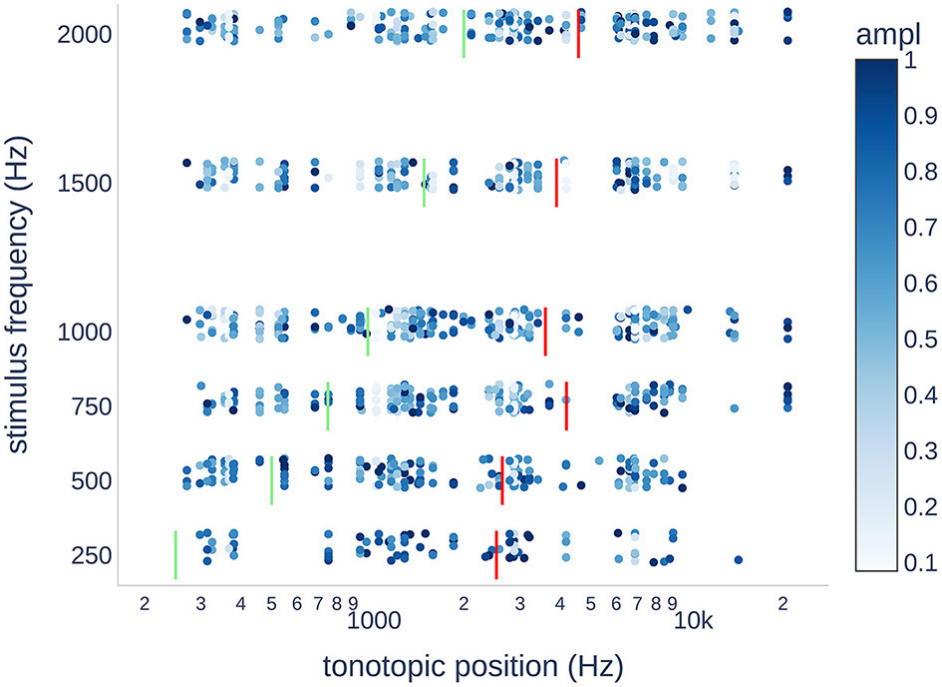
Scatter plot of the normalized CM/DIF response amplitudes for each stimulus frequency. The expected tonotopic position is shown in green. The weighted mean tonotopic position found is shown in red. In terms of the tonotopic position, there was a large variance in the objectively present ECochG responses. Generally, the tonotopic position was found to be higher than the stimulation frequency by up to an order of magnitude. Outliers at 20,677 Hz were all from PO19.

**Figure 7:**
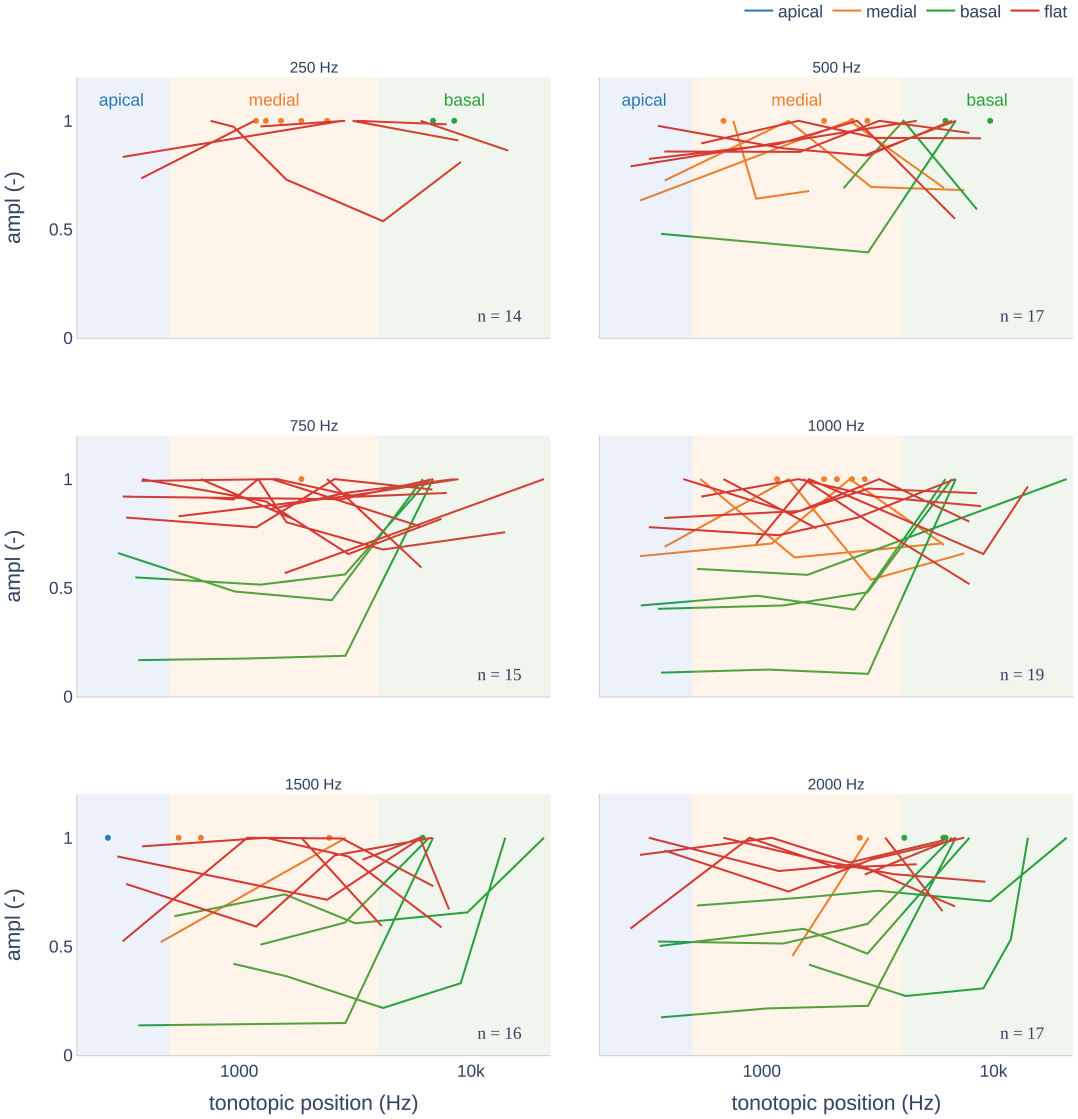
CM/DIF pattern distributions recorded from pure-tone stimuli. Depending on the cochlear frequency regions, we distinguished between an apical, medial, and basal peak. If the ECochG amplitudes were approximately the same over the entire electrode array, this was referred to as a flat pattern.

**Figure 8:**
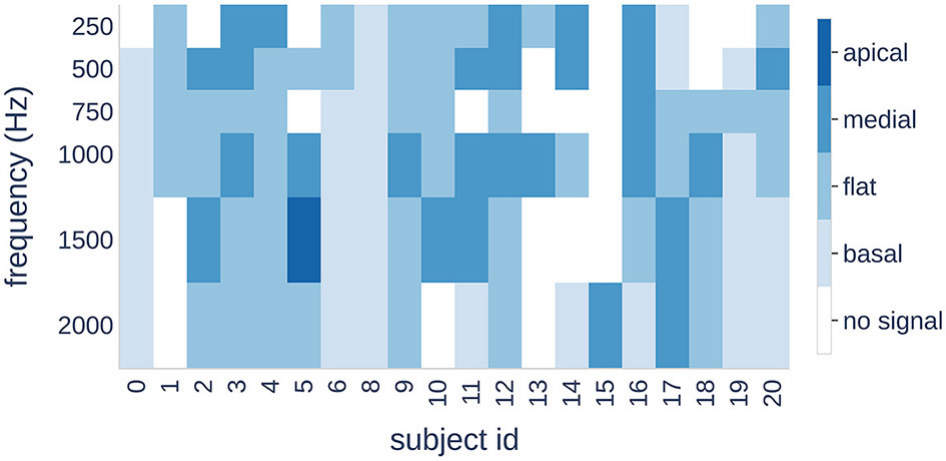
Four patterns (color bars) are shown for each subject (horizontal axis) and each stimulus frequency (vertical axis). A change in color indicates a shift of the maximum signal amplitude to a different cochlear region.

Regarding the intracochlear amplitude distributions, Figure 7 shows the normalized amplitude as a function of the tonotopic and stimulus frequencies. We found a predominance of flat patterns, occurring in 44 cases, followed by medial and basal, each occurring in 27 and 26 cases, respectively. The least common pattern was apical, occurring in only one case. Otherwise, all stimulation frequencies were observed for all the other patterns. However, the basal pattern was more pronounced at frequencies >500 Hz. Additionally, for each subject, we examined whether there was a change in the amplitude pattern as a function of the stimulation frequency. Figure 8 shows the patterns observed for each subject for each stimulus frequency, respectively. For example, subject PO8 showed the same CM/DIF response pattern for all stimulation frequencies (i.e., basal pattern; amplitude maxima for all frequencies occurred in the basal part of the cochlea). In contrast, subject PO16 showed a more dynamic pattern with the amplitude maxima changing from medial (250–1,000 Hz) to flat (1,500 Hz) to apical (2,000 Hz).

## 4. Discussion

In this study, we used an objective DL-based algorithm to evaluate intracochlear, post-operative ECochG signals recorded three times over a period of ~3 months. The use of an objective algorithm has several advantages, for instance, the data are analyzed independently of experts and always in the same manner. Regardless of the SNR, all the data were included in the analysis, which prevented selection and reporting bias. Finally, our algorithm is open-access, which makes the analysis transparent (18). Therefore, we were able to study and compare cross-sectional and longitudinal ECochG data systematically in the first place. In our analysis, we used the following three research questions: Are the recordings longitudinally reproducible?; is there a correlation with the pure-tone threshold?; and can we detect patterns for stimuli of different frequencies?

### 4.1. Repeatability

Our results showed substantial repeatability of the CM/DIF responses over the three measurement sessions (PABAK 0.68, accuracy of 83.8%) (31). This result is comparable to other neurophysiological findings, such as waving the V responses in the auditory brainstem measurements (36, 37). Analysis of the combination of two sessions showed a higher PABAK value for sessions 1 and 2 (0.74) than for sessions 1 and 3 (0.65) and 2 and 3 (0.66). This could also be shown statistically, where there was only a significant difference between sessions 1 and 3, and sessions 2 and 3, but not between sessions 1 and 2. A possible explanation for this finding is the altered measurement conditions, such as a change in the eartip placement, which could reduce the presented intensity level of the acoustic stimulus (38). However, a random effect without a clear pattern was expected in this case. Therefore, we suspect that we were detecting a discrete longitudinal change in the inner ear function although the hearing thresholds were unchanged between sessions 1 and 3. It is well-known that pure-tone audiometry cannot detect small changes in hearing and is prone to variability (39). Additionally, other studies have shown that the inner ear function of CI users declines over the years (19, 40). This decline may be caused by the natural course of the inner ear disease or by slowly progressive cochlear fibrosis as part of the immune response to the electrode array (41). However, the assumption that ECochG can reliably detect discrete inner ear changes should be confirmed in follow-up studies.

Figure 6 shows the normalized CM/DIF amplitudes for all subjects and stimulus frequencies. The weighted mean of the CM/DIF responses (represented by red bars) was substantially higher than the expected tonotopic position (represented by green bars). Furthermore, we observed signals in all the tonotopic regions for each stimulus, with a large variance in the data points. We found a moderate relationship between the stimulus frequency and weighted means of the CM/DIF responses (*r*^2^ = 0.70, *p* = 0.039). The mean tonotopic positions for the 250 and 500 Hz stimuli were located in the medial cochlear region, whereas the mean tonotopic positions for the higher frequency stimuli were located in the medial and basal regions.

In our results, PABAK values were the highest for the sub-threshold stimulation and the lowest for the near-threshold stimulation. This is not surprising because small variations can lead to a CM/DIF response being detected (or not) by the algorithm. It should be noted that the supra-threshold group contained fewer values because not all subjects had a hearing threshold that allowed >25 dB stimulation at all frequencies. Therefore, the supra-threshold group showed an increased variance. Additional data are needed to confirm this point.

As described by other researchers (9, 11, 12, 15, 16, 42) and our findings, ECochG recordings show a large, individual variance of amplitudes (4.56 μV_pp_ to 74.46 μV_pp_). Small amplitudes in poor SNR situations may not be detected by the algorithm. The proposed open-access algorithm can be continuously improved in the future. Therefore, future refinement may improve this resolution.

### 4.2. Thresholds

In our data, we found a moderate to strong dependence between the CM/DIF and audiometric thresholds. These results are consistent with those of previous research and suggest that post-operative CM/DIF thresholds can be used as objective markers for estimating residual hearing (9, 10). Overall, higher relative stimulation levels resulted in a greater number of objectively detected CM/DIF responses. The mean relative stimulation level that elicited the response was 14.1 dB. Additionally, there was a large variance in the relative hearing threshold that elicited an ECochG response. In some cases, stimuli that were 30 dB below the hearing threshold elicited an inner ear response (see Figure 3). Overall, the use of below-threshold stimuli resulted in detectable responses in 11.6% of the cases. However, the CM/DIF responses were not always elicited at stimulus levels well above the hearing threshold (up to 48 dB). These results were described in previous studies (10, 16, 42, 43).

The mean ECochG thresholds were above the pure-tone thresholds for all stimulus frequencies. Other groups stated that compared to the pure-tone audiogram, the ECochG threshold was overall lower (16, 43) or higher at lower frequencies and lower at higher frequencies (9, 10). When comparing the results from other studies, it is important to note that the study design may differ (e.g., measurement hardware and software, stimulation protocol, and the use of different scales dB nHL). Recordings were also made at the most apical electrode, whereas we chose the electrode with the lowest CM/DIF threshold (9, 10, 16, 43). Additionally, analysis were performed differently. The signals were evaluated visually (16) or a binning method using the FFT spectrum (4). In one case, the total signal was calculated (adding the SUM and DIF responses) and used instead (43). It should also be noted that some studies compared post-insertion ECochG thresholds with post-operative audiograms (16, 43). However, the residual hearing may have decreased during this period (19, 40).

A subjective loudness scale (instead of pure-tone thresholds) showed a strong correlation for all groups. As expected from the data in Table 2, the correlation was the highest for the *very soft* and *soft* groups. For the *cannot hear* and *medium* groups, we found more outliers reducing utility of a linear model. Outlier in the *cannot hear* group were mostly recorded at higher stimulus frequencies. Therefore, we assume that the subjects heard the repetition rate of the acoustic stimuli and not the actual stimulus frequency.

### 4.3. Tonotopy and patterns

With respect to the maximum signal amplitudes, we observed a tendency toward tonotopic allocation in our data. However, there was a large variance and the classification was not applicable to all study subjects. Published studies have shown that intracochlear ECochG amplitudes increase toward the tonotopic generator (13, 15, 17). However, some patterns did not follow this order (11–13, 17, 43). It should also be noted that intracochlear ECochG recordings were analyzed using electrode numbers but not the tonotopic locations of the measuring points (9, 11–13, 17). In our opinion, this approach is not optimal. Depending on the study, different electrode arrays (with corresponding variations in the length and inter-electrode spacing) were used. Additionally, the length of the cochlear duct can vary significantly, affecting the tonotopic position (35). Radiographic specification of the tonotopic position may be regarded as more accurate. If available, this information can be obtained using postoperative CT scans. If not available, impedance values or average insertion depths can be used to estimate the electrode positions (44). A possible explanation for the failure to maintain tonotopic organization could be the differences in the function of the hair cell segments within the cochlea. This may result in signal generators that lead to a divergence in the signal pattern (11–13, 17, 43).

In the present study, we observed a clear basal shift in the tonotopic allocation. When stimulated at 250 Hz, the weighted mean was ~2.5 kHz. This tendency increased when stimulated at higher frequencies. Thus, a 2 kHz stimulus resulted in a weighted mean at ~4.6 kHz (see Figure 6). There are several possible explanations for basal shift. High-intensity stimuli can activate basally located hair cell populations (3, 11, 15). Additionally, the electrode can touch the basilar membrane and alter the mechanical properties of the microstructures involved in the transduction process (e.g., increased stiffness) (12, 45). Similarly, trauma to the basilar membrane or intracochlear fibrosis as a result of the introduced foreign body could result in a deviation of the stimulation characteristics with a corresponding frequency shift (3, 46).

We divided the CM/DIF amplitudes into four patterns similar to those described in previous research (11, 12, 14, 17). Hypothetically, for a 500 Hz stimulus, we expected a maximum peak in the 500 Hz region (according to our frequency subdivision at the border of the apical and medial cochlear segments). In our study population, the flat pattern was the most common. This finding is consistent with those of Bester et al. (11, 12). One can only speculate on the reasons for the missing peaks. It is possible that poorly functioning hair cell populations are responsible for this phenomenon. If this pattern is already present at the time of electrode insertion, it would certainly be relevant. Many authors expect an apical peak to occur under intra-oberative conditions. Traumatic inner ear events are often suspected in the case of a drop. If a subject does not have a peak pattern (but rather a flat pattern or a basal peak), the CM/DIF amplitude will not increase or even decrease, and the surgeon may be misled into assuming an intracochlear traumatic event. Furthermore, we found that when a peak pattern was present, the tonotopic position of the peak was rarely congruent with the stimulation frequency. A basal shift in the peak patterns was observed with increasing frequency. At 1.5 and 2 kHz, the peaks were not located in the basal region but rather in the medial segment of the cochlea. Bester et al. described this a basal shift when ECochG recordings were repeated after 3 months (12). This could also explain our results because our data were recorded at least 6 months after implantation.

Finally, we examined the individual distribution of the patterns in response to different stimulus frequencies. Our results showed that three subjects had the same pattern for all frequencies, whereas the other subjects showed a transition from one pattern to another.

In conclusion, amplitude patterns can provide important information regarding inner ear function with the implant electrode in place. Further data analysis is necessary to determine which factors are responsible for these patterns.

### 4.4. Limitations

Our study population had relatively low residual hearing with a mean PTA of 88.7 dB HL (Table 1). However, we were able to measure the ECochG response over time in all but one subject. In our analysis, we focused on the CM/DIF signals. In future, other signal subtypes should be addressed (e.g., ANN/SUM, CAP, SP potentials, latency measures) (47). These signal subtypes can be implemented using an improved DL algorithm. Finally, other intracochlear biomarkers should be included in the analysis (e.g., impedance measures) as they may reflect around the electrode carrier (12, 48, 49).

## 5. Conclusions

In this study, we successfully implemented an objective DL-based algorithm to evaluate post-operative intracochlear ECochG recordings. Using an objective analysis, we systematically evaluated and compared ECochG data. In our study, CM/DIF recordings showed substantial repeatability and may indicate the feasibility of using ECochG to monitor inner ear health over time. Additionally, the CM/DIF thresholds showed moderate to strong correlations with audiometric and subjective hearing levels. Finally, we found a basal shift in the tonotopic position of the CM/DIF responses as well as specific intracochlear peak patterns.

Our results help to identify signal patterns and thus better understand inner ear functions with the electrode in place. As a next step, the algorithm should be applied to intra-oberative recordings.

## Data availability statement

The datasets presented in this study can be found in online repositories. The name of the repository and accession number can be found at: Dryad, https://datadryad.org/stash/share/hE4oniHffFhggCJzN36T9QnwMqw79nBMeo3A1WNsW3s.

## Ethics statement

The studies involving human participants were reviewed and approved by the Cantonal Ethics Committee of Bern (BASEC ID 2019-01578). The patients/participants provided their written informed consent to participate in this study. Written informed consent was obtained from the individuals for the publication of any potentially identifiable data included in this article.

## Author contributions

KS performed the measurements, analyzed the data, and wrote the software and paper. WW, CR, and MC provided interpretive analysis and critical revision. SW designed the experiment, analyzed the data, and provided interpretive analysis and critical revision. All authors contributed to this work.
