# Supplementary figures and images for: Objective evaluation of intracochlear electrocochleography: repeatability, thresholds, and tonotopic patterns
Source: Front Neurol. 2023 Aug 8;14:1181539. doi: 10.3389/fneur.2023.1181539 (PMC10446839; doi:10.3389/fneur.2023.1181539)

## Audiogram

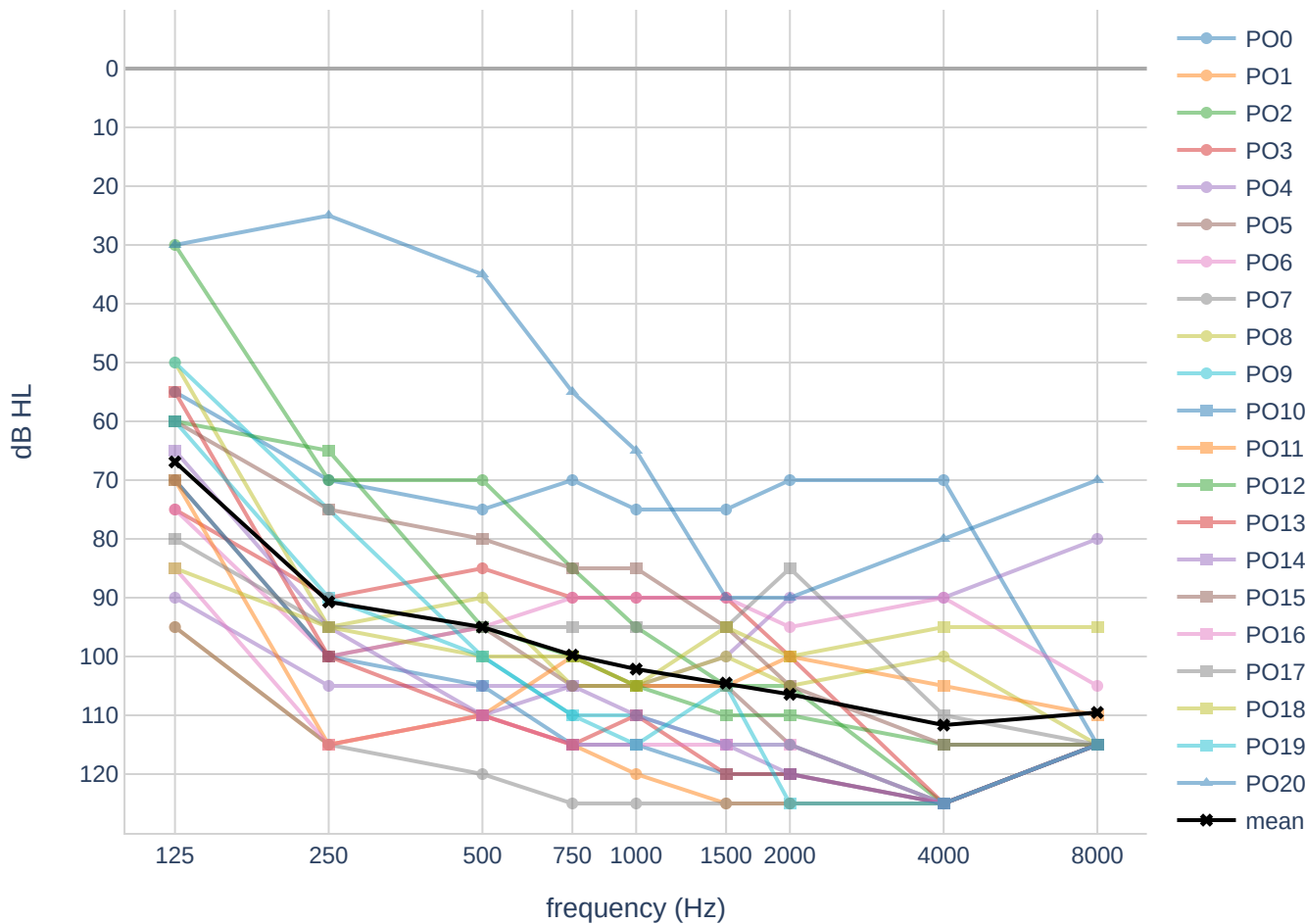

Supplement: Supplementary file 2 [file Data_Sheet_1.PDF]
