# Supplementary material for: Objective evaluation of intracochlear electrocochleography: repeatability, thresholds, and tonotopic patterns
Source: Front Neurol. 2023 Aug 8;14:1181539. doi: 10.3389/fneur.2023.1181539 (PMC10446839; doi:10.3389/fneur.2023.1181539)
Supplement: Supplementary file 3 [file Data_Sheet_2.PDF]

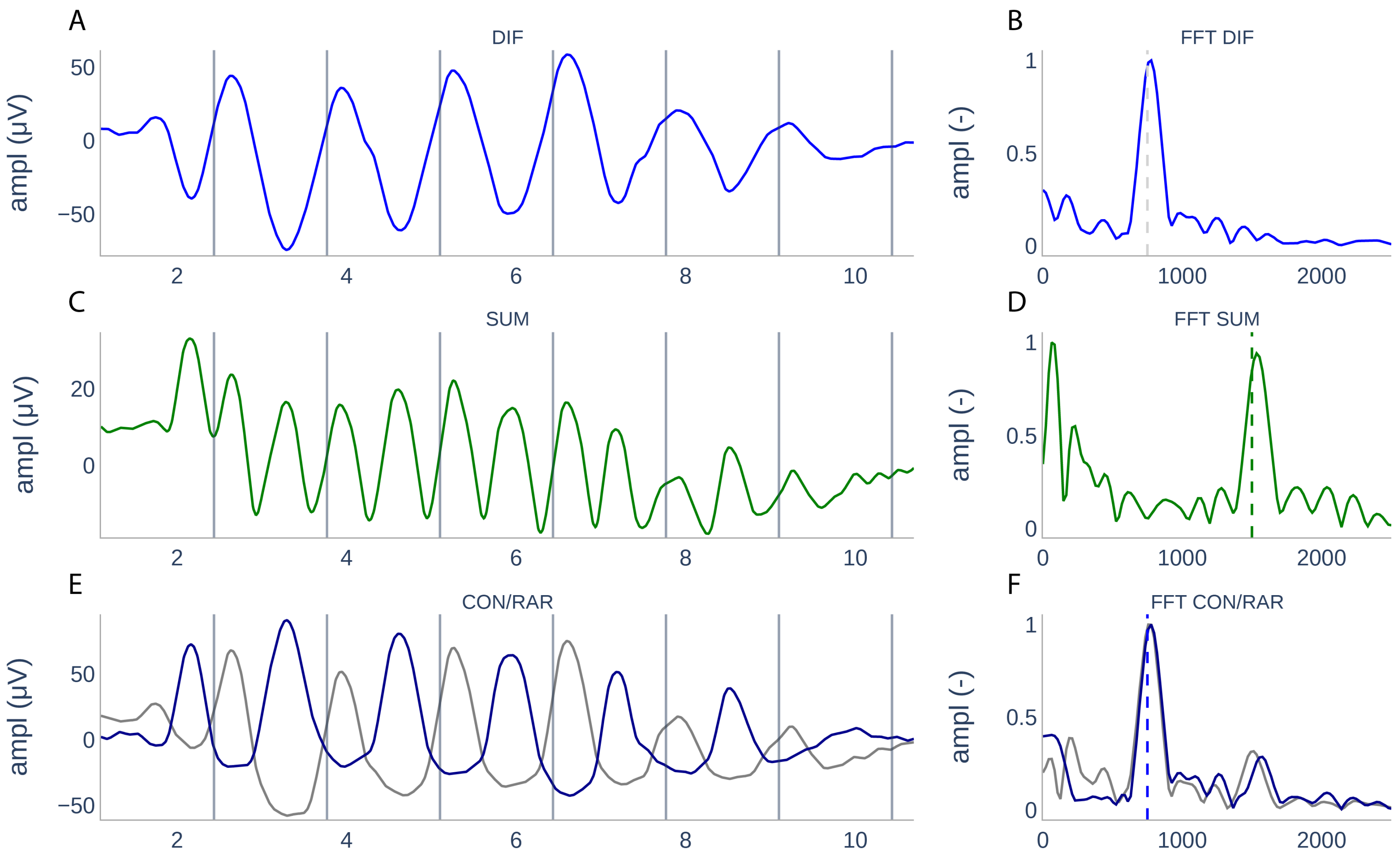

Example recording from subject PO8 of A) CM/DIF, C) ANN/SUM, E) CON and RAR, and B,D,F) their individual FFT power spectra
